# Multitasking Compensatory Saccadic Training Program for Hemianopia Patients: A New Approach With 3-Dimensional Real-World Objects

**DOI:** 10.1167/tvst.10.2.3

**Published:** 2021-02-05

**Authors:** Laura Mena-Garcia, Jose C. Pastor-Jimeno, Miguel J. Maldonado, Maria B. Coco-Martin, Itziar Fernandez, Juan F. Arenillas

**Affiliations:** 1 Instituto Universitario de Oftalmobiología Aplicada (IOBA), Eye Institute, Universidad de Valladolid, Valladolid, Spain; 2 Universidad de Valladolid, Valladolid, Spain; 3 Department of Ophthalmology, Hospital Clínico Universitario de Valladolid, Valladolid, Spain; 4 Red Temática de Investigación Colaborativa en Oftalmología (OftaRed), Instituto de Salud Carlos III, Madrid, Spain; 5 Department of Neurology, Hospital Clínico Universitario de Valladolid, Valladolid, Spain; 6 Biomedical Research Networking Center in Bioengineering, Biomaterials and Nanomedicine (CIBER-BBN), Valladolid, Spain

**Keywords:** hemianopia, neuroplasticity, compensatory saccade training, eye-hand coordination, neurovisual rehabilitation

## Abstract

**Purpose:**

To examine whether a noncomputerized multitasking compensatory saccadic training program (MCSTP) for patients with hemianopia, based on a reading regimen and eight exercises that recreate everyday visuomotor activities using three-dimensional (3D) real-world objects, improves the visual ability/function, quality of life (QL), and functional independence (FI).

**Methods:**

The 3D-MCSTP included four in-office visits and two customized home-based daily training sessions over 12 weeks. A quasiexperimental, pretest/posttest study design was carried out with an intervention group (IG) (*n* = 20) and a no-training group (NTG) (*n* = 20) matched for age, hemianopia type, and brain injury duration.

**Results:**

The groups were comparable for the main baseline variables and all participants (*n* = 40) completed the study. The IG mainly showed significant improvements in visual-processing speed (57.34% ± 19.28%; *P* < 0.0001) and visual attention/retention ability (26.67% ± 19.21%; *P* < 0.0001), which also were significantly greater (*P* < 0.05) than in the NTG. Moreover, the IG showed large effect sizes (Cohen's d) in 75% of the total QL and FI dimensions analyzed; in contrast to the NTG that showed negligible mean effect sizes in 96% of these dimensions.

**Conclusions:**

The customized 3D-MCSTP was associated with a satisfactory response in the IG for improving complex visual processing, QL, and FI.

**Translational Relevance:**

Neurovisual rehabilitation of patients with hemianopia seems more efficient when programs combine in-office visits and customized home-based training sessions based on real objects and simulating real-life conditions, than no treatment or previously reported computer-screen approaches, probably because of better stimulation of patients´ motivation and visual-processing speed brain mechanisms.

## Introduction

Search-and-reach tasks of three-dimensional (3D) real-world objects in a changing visual environment are crucial to the efficient performance of everyday multitasking activities.^1^^,^^2^ Multitasking refers to the execution of two or more tasks/executive brain processes performed in the same time, either simultaneously (parallel-brain information processing) or in rapid succession (serial-brain information processing).^3^^,^^4^ Overt and covert visual attention networks are key to appropriate control of visuomotor systems and efficient performance of everyday multitasking activities.^2^^,^^5^ The lack of peripheral vision in patients with homonymous visual field defects, such as the hemianopia or quadrantanopia type in 30% to 85% of patients with acquired brain injury,^6^ are associated with lower activation of their attention brain mechanisms^7^^,^^8^ and a tendency to scan visual scenes using more frequent fixations and shorter saccades than visually normal controls.^9^ Moreover, it has been shown objectively that patients with homonymous visual field defects have 73% lower visual-processing speed than healthy controls.^10^ Visual-processing speed is a quantifiable parameter of visual ability based on measurements of reaction time, which depend on the proper functioning of six main brain-processing systems: attentional, visuocognitive, visuomotor, working memory, auditory-cognitive, and executive.^5^^,^^11^^–^^13^ Subsequently, patients with homonymous field defects have difficulty performing daily activities requiring activation of parallel and serial-brain mechanisms, such as reading, orientation, mobility, depth perception, and eye-hand coordination tasks.^14^^,^^15^ Therefore homonymous field defects are considered important disabilities associated with frustration and insecurity that reduce significantly patients’ quality of life (QL) and functional independence (FI).^14^^,^^15^

The scientific community has joined efforts to develop effective neurovisual rehabilitation training programs for patients with homonymous visual field defects. Daily home-based computerized compensatory training programs^15^^,^^16^ are widely used approaches to improve the quality of ocular movements,^17^ reading performance,^18^ and searching reaction time.^17^^,^^18^ These programs are based on brain-plasticity theories^19^ and principally combine computerized visual-searching tasks of low complexity stimuli (lights or simple 2D images, e.g., symbols, letters, or numbers) on a computer screen (2D environment), with other specific attention and computerized reading exercises, over a maximal training period of six weeks. However, publications about the effectiveness of previous compensatory neurovisual rehabilitation training regimens have suggested two main limitations, that is, the lack of eye-hand coordination exercises might be one factor contributing to small improvements in the QL after training,^15^ and the fact that reaction time evaluation methods are equal or similar to those of training^17^^,^^18^ could contribute to possible biases in the final results due to the learning effect phenomenon. Finally, recent cognitive neurology studies have shown that 3D real-world objects elicit stronger action-related brain responses than 2D images,^20^^–^^22^ even in patients with visual agnosia.^23^

Nevertheless, national health systems worldwide generally lack specialized neurovisual rehabilitation units.^24^^–^^26^ Consequently, once the neurologic and ophthalmologic injuries are stabilized in patients with hemianopia, they are discharged from national health systems without the option of a customized neurovisual rehabilitation program, and subsequently they have to manage the new visual condition on their own.

Thus the main purpose of this study was to demonstrate whether a non-computerized multitasking compensatory saccadic training program (MCSTP) improved visual ability/function, QL, and FI in patients with hemianopia compared to a no-training group (NTG). We have also tried to overcome the principal limitations of the existing computerized compensatory trainings, recreating everyday visuomotor activities using 3D real objects. In addition, to avoid biases related to the learning effect visual ability/function assessment tests different from the training ones were used.

## Methods

All procedures were performed in accordance with the Declaration of Helsinki.^27^ The local clinical research ethics committee approved the study protocol, code PI-13-126 CINV-13-46. All candidates provided written informed consent.

### Participants and Study Design

Seventy-four patients with chronic homonymous visual field defects from any postchiasmatic acquired brain injury were referred from four local clinical centers in Valladolid, Spain (Instituto Universitario de Oftalmobiología Aplicada [IOBA]–Eye Institute, Hospital Clinico Universitario, Hospital Universitario Rio Hortega, and ICTIA [Association of Strokes and Paresis of Castilla y León]) to the IOBA-Eye Institute Neurorehabilitation Unit. The participants had to be at least 18 years old and should not have undergone a neurovisual rehabilitation training previously. Additional inclusion criteria included total neuro-ophthalmologic, neurologic, and radiologic clinical stability (≥3 months after the acquired brain injury to minimize confounding by spontaneous recovery^28^); measurement of the distance best-corrected binocular visual acuity ≥0.1 logarithm of the minimum angle of resolution visual acuity using the Early Treatment Diabetic Retinopathy Study Chart; no visual hemineglect based on the clock-drawing^29^ and line-bisection tests,^30^ visual agnosia based on the Poppelreuter-Ghent test^31^ or cognitive deficit based on the Mini-Mental State Examination^32^; and sufficient residual hearing and hand-arm ability to complete the training without special assistance.

The 3D-MCSTP was carried out through a quasi-experimental, pretest-posttest study design,^33^^–^^36^ with an intervention group (IG) (*n* = 20) and a NTG (*n* = 20) matched for age, hemianopia type, and brain injury duration. The NTG participants were offered the 3D-MCSTP at the end of the study.

### Experimental Intervention: Scientific Basis, Materials, and Procedures of the Noncomputerized 3D-MCSTP

**Figure 1.**
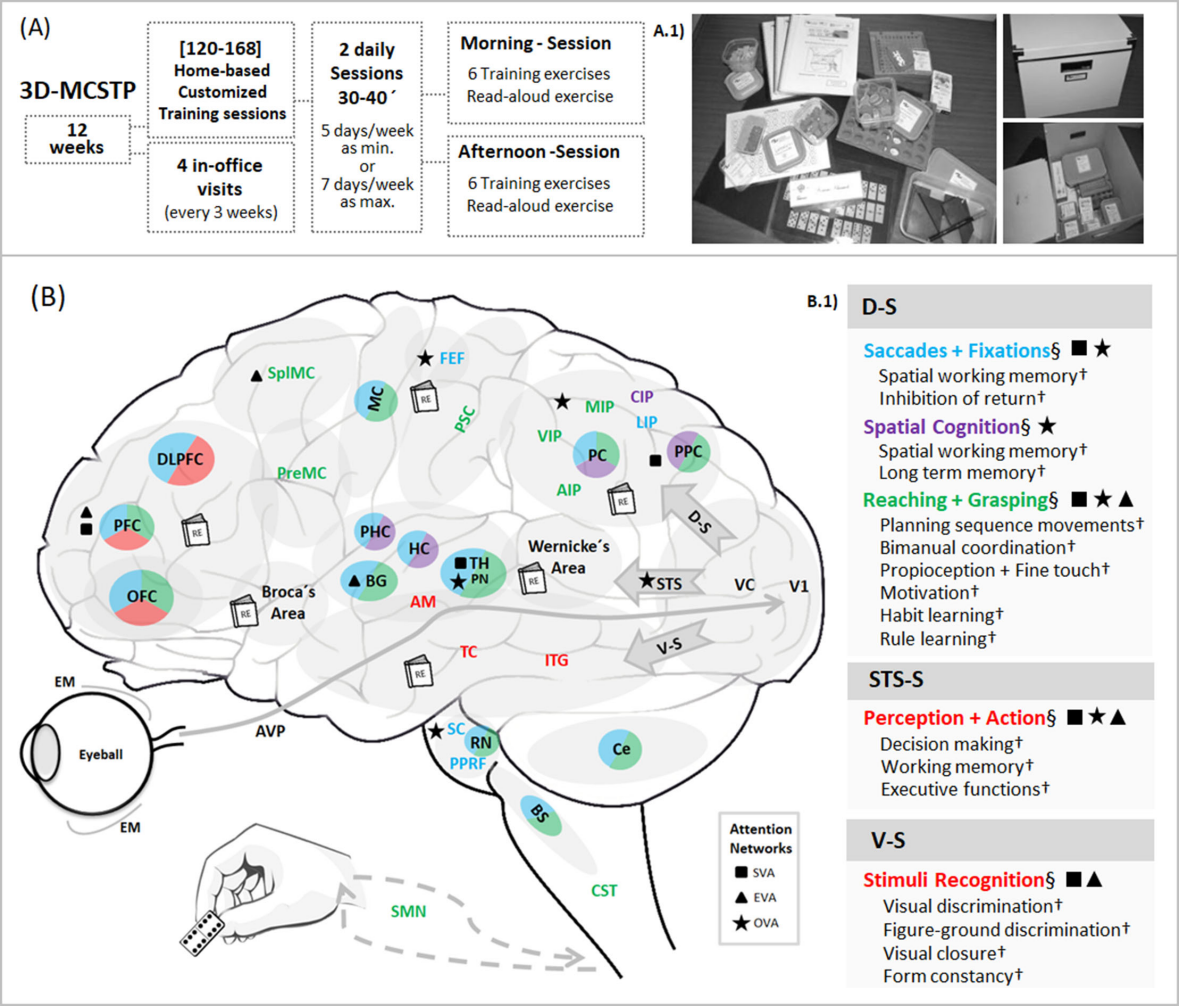
The 3D-MCSTP. (A) Schematic diagram of the intervention group training protocol. (A.1) Pictures of the 3D-MCSTP materials pack. (B) This graph was developed on the basis of primate studies and human neuropsychology data already published.^37^^,^^44^^–^^54^ It summarizes the main visual brain structures involved in the performance of six daily visuocognitive and visuomotor behaviors (§B.1); corresponding colors). The *gray arrow* indicates the afferent visual pathway (*AVP*) from the eyeball to the *V1* area of the occipital lobe. The three *thick arrows* indicate the beginning of the three main secondary visual pathways involved in the visual processing: dorsal stream (*D-S*), ventral stream (*V-S*), and superior temporal sulcus stream (*STS-S*). The book drawings represent the main brain areas involved in the read-aloud exercise (*RE*). (B.1) Schematic summary with the three secondary visual pathways, their corresponding six main visuocognitive and visuomotor behaviors, and †their main associated attentional and executive abilities, which correspond to those that patients had to put into play to correctly perform the training program at home. AIP, anterior intraparietal area; AM, amygdala; BG, basal ganglia; BS, brainstem; Ce, cerebellum; CIP, caudal intraparietal area; CST, corticospinal tract; DLPFC, dorsolateral prefrontal cortex; EM, extraocular muscles; EVA, executive visual attention; FEF, frontal eye fields; HC, hippocampus; ITG, inferotemporal gyrus; LIP, lateral intraparietal area; MC, medial cortex; MIP, medial intraparietal area; OFC, orbitofrontal cortex; OVA, orienting visual attention; PC, parietal cortex; PFC, prefrontal cortex; PHC, parahippocampus; PN, pulvinar nuclei; PPC, posterior parietal cortex; PPRF, paramedian pontine reticular formation; PreMC, premotor cortex; PSC, primary somatosensory cortex; RN, red nuclei; SC, superior colliculus; SMN, spinal motor neurons; SplMC, supplementary motor cortex; SVA, sustained visual attention; TC, temporal cortex; TH, thalamus; VC, visual cortex; VIP, ventral intraparietal area.

Humans can only create new and stable neural connections by repeating actions over time.^37^ Previous studies have established 12 weeks as the minimal training period to improve a patient's brain attention networks.^38^^–^^40^ Furthermore, daily practice and motivation seem to be decisive factors in improving the use of residual vision in subjects with impaired vision.^18^^,^^41^^–^^43^ Thus the 3D-MCSTP comprises four in-office visits (every three weeks) and two customized home-based daily training sessions (30 to 40 minutes in the morning and afternoon) that should be performed a minimum of five days/week or a maximum of seven days/week, over 12 weeks. In other words, the 3D-MCSTP facilitated a minimum of 120 and a maximum of 168 home-based training sessions during the time interval between in-office visits (Fig. 1A).

**Figure 2.**
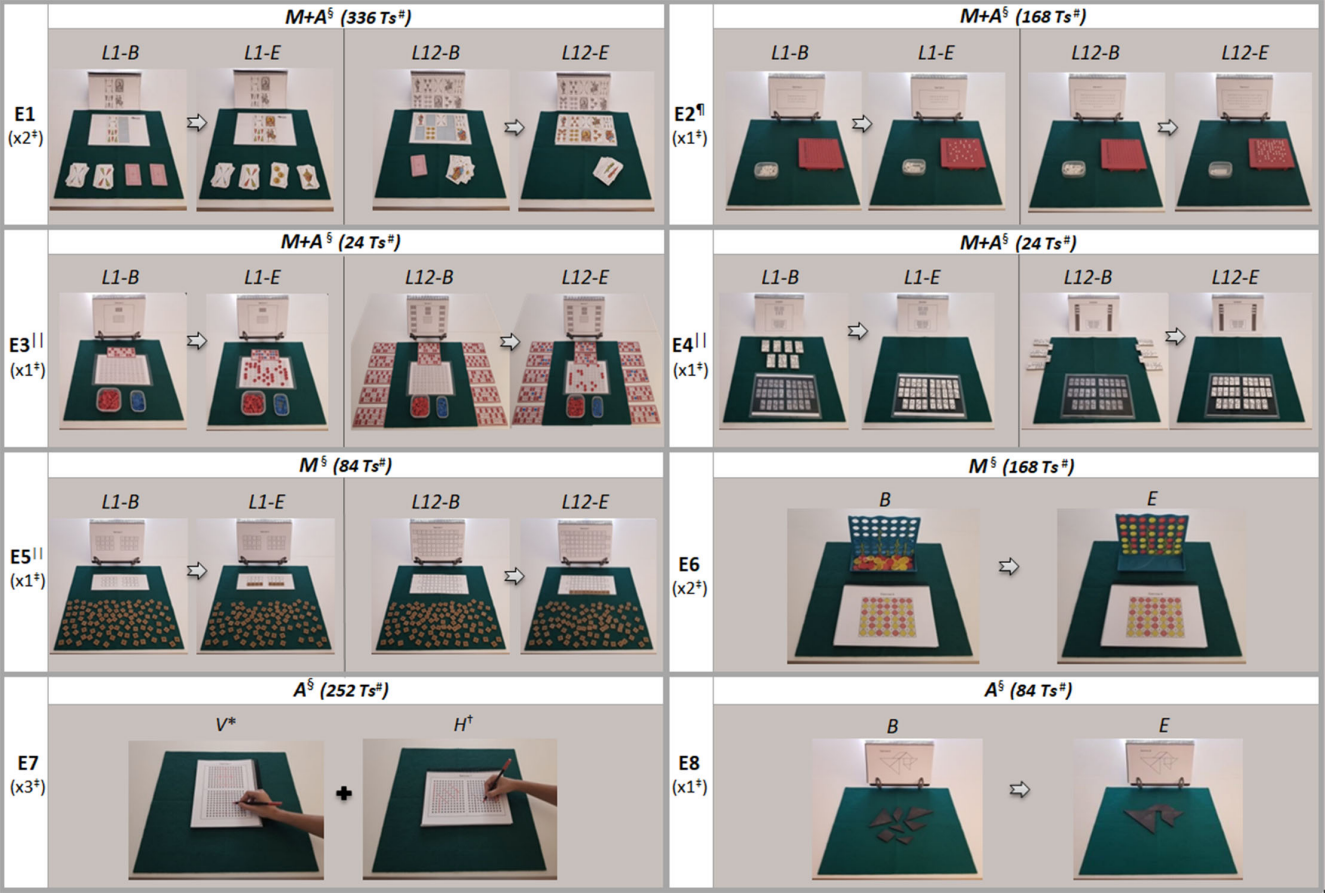
Examples of each included exercise (E) of the 3D-MCSTP. The E1–E5 images show the levels from lower (level [L] 1) to higher difficulty (L 12) at the beginning (*B*) and at the end (*E*) of the exercise. The images of E6 and E8 show the B and the E of the exercise because there were no pre-established levels of difficulty for them. E7 displays the two types of pattern copy, vertical (*V*) and horizontal (*H*). In all the images there is a green card mat that serves as a reference when properly placing the training materials on the worktable. ^*^Patients should do one vertical sheet at each training session. In addition, the copy pattern alternates its position between the top and the bottom of the sheet every day. ^†^Patients should do two horizontal sheets at each training session, and, in this case, the copy pattern position is customized according to the patient´s visual field defect to the right or to the left of the sheet. ^‡^The number of training sheets that a patient had to complete in each daily session. ^§^It is specified whether the patients had to perform the exercise in the morning session (*M*), in the afternoon session (*A*), or in both (*M + A*). ^#^ The total number of different training sheets incorporated in the 3D-MCSTP for each type of exercise to avoid learning effect. ^¶^The 10 × 10 grid position was customized according to the patient's visual field defect. ^||^The positions of the bingo cards (E3), dominoes (E4), and the 100 plastic pieces with letters (E5) changed positions between the top and bottom of the card mat between training sessions.

On the basis of theories of cognitive neuroscience and visual brain neuroplasticity after an acquired brain injury,^24^ it was considered essential for patients with hemianopia that the 3D-MCSTP performance could stimulate the main neuronal visual structures (afferent visual pathway, dorsal stream, ventral stream, and superior temporal sulcus stream) involved both in reading tasks and in visuocognitive and visuomotor daily life behaviors and their main associated attentional and executive abilities (Figs. 1B, B.1). ^37^^,^^44^^–^^54^ Accordingly, and based on previous studies that only found reading performance improvements when a reading task was specifically trained through a daily neurovisual rehabilitation regimen,^15^^,^^55^ the 3D-MCSTP included a minimum of 10 minutes of daily home-based read-aloud exercises. Patients chose a book they wished to read, which should be motivating, with a font size of 12 points or larger. Additionally, to recreate everyday visuomotor activities based on the execution of two or more tasks/executive brain processes in the same time window in rapid succession (serial-brain information processing),^3^^,^^4^ eight different types of multitasking exercises were included in each home-based daily training regimen. Subsequently, in general terms, these exercises were based principally on search-and-reach tasks that should be performed in the shortest possible time and without head movements by means of serial-brain information processing: saccadic eye movements and fixations, spatial cognition, arm movements for reaching, hand movements for grasping, stimuli recognition, and coordinated perception and action brain mechanisms (Figs. 1B, B.1). Thus the main materials selected for designing the eight multitasking exercises were 3D real-world objects that are part of seven international table games (Fig. 2 and Supplementary Material A).

A total of 1140 different training sheets and 96 instruction sheets with information about how to perform each exercise properly were developed and grouped into four neurovisual rehabilitation notebooks. To recreate a changing visual environment, exercises 1 to 6 consisted overall of searching one by one for a specific 3D real-world object among a set of ones designed to distract (Fig. 2). Once the required 3D real-world object was found, it had to be reached for, grasped, moved, and positioned at the specific worktable position required by the training or instruction sheets, in such a way that the patient´s visual environment remained in constant change. Exercises 7 and 8 consisted of pattern copy exercises, which required decision-making actions, because patients decided how to perform the exercise to finish them in the shortest possible time (Fig. 2).

**Figure 3.**
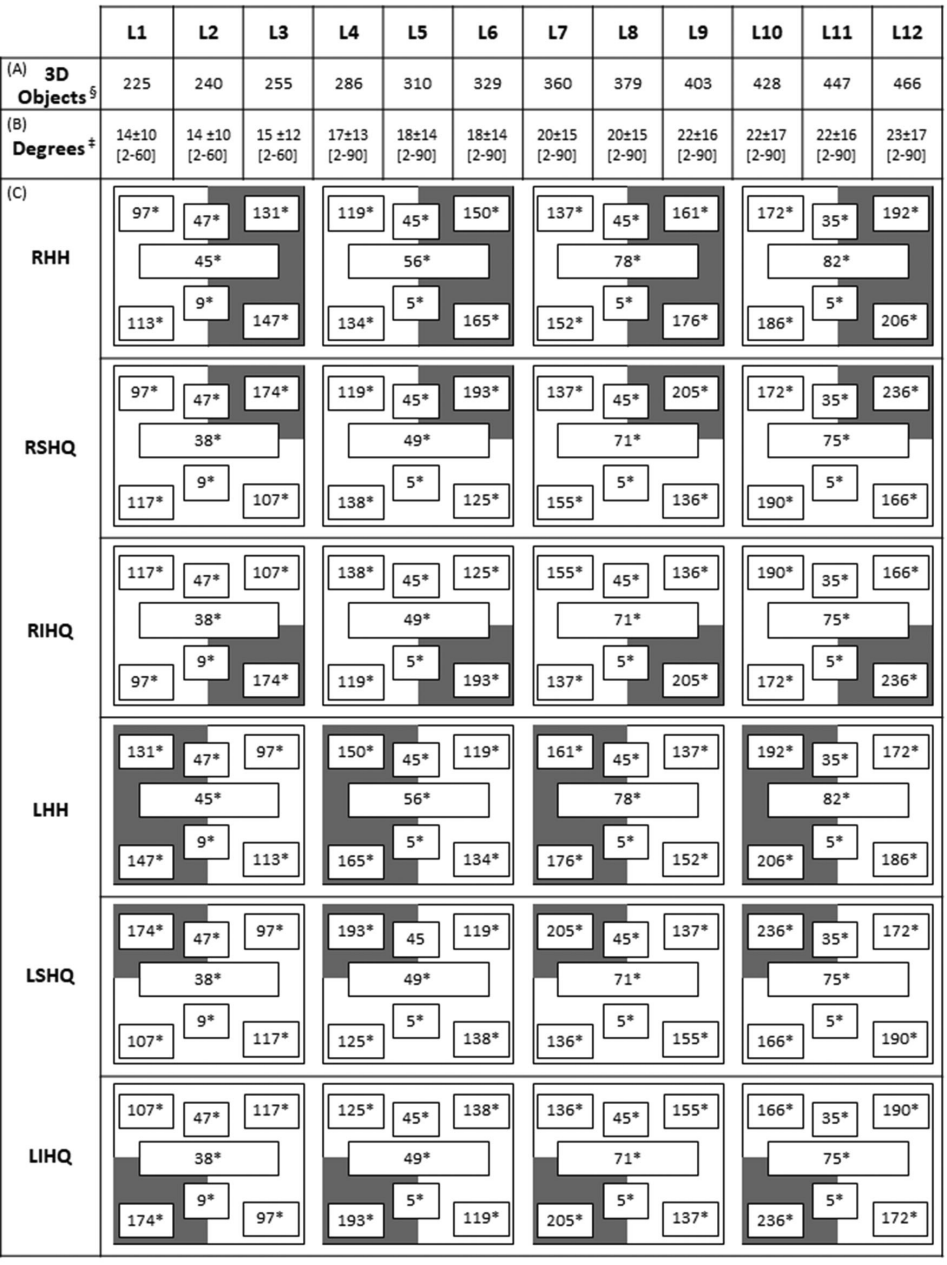
The schematic summary of the six different customized versions of the 3D-MCSTP according to the six more prevalent types of hemianopia and quadrantanopia. Each version had 12 common levels (Ln) of difficulty based on increases level by level of the variables. (A) ^§^The total number of 3D real-world objects. (B) ^‡^The number of trained horizontal degrees (mean ± SD) (range). (C) *The total visual distracting stimuli located on the worktable at seven spatial locations (gray indicates the blind field and white the seeing field). Note: All included values correspond to the mean value obtained by considering both daily training sessions. RHH, right homonymous hemianopia; RSHQ, right superior homonymous quadrantanopia; RIHQ, right inferior homonymous quadrantanopia; LHH, left homonymous hemianopia; LSHQ, left superior homonymous quadrantanopia; LIHQ, left inferior homonymous quadrantanopia.

Furthermore, based on the fact that the visual-processing speed and attention system's activation will be greater with shorter, newer, and complex tasks,^5^^,^^11^^–^^13^ each of the eight neurovisual rehabilitation exercises were intended to be completed in a maximal time of five minutes. Moreover, 12 difficulty levels were created for each exercise and the following three variables increased level by level: the total number of 3D real-world objects that patients had to reach to grasp and place in the correct area on the worktable (Fig. 3A); second, the number of horizontal degrees (to the right and left of the patient's visual field) along which patients had to search for, reach, grasp, and place stimuli (Fig. 3B); and third, the total visual-distracting stimuli (drawings, letters, numbers, etc.) on the worktable at seven spatial locations of the patient's visual field (Fig. 3C). However, depending on the type of homonymous visual field defect, the highest number of visual-distracting stimuli was always at the spatial location matched with the blind visual field. Thus, for the first time, six different versions within the same neurovisual rehabilitation training program were developed to customize the exercises for each patient according to the six more prevalent types of homonymous visual field defects (Fig. 3C).

Finally, to assess the level of compliance of the training regimen and avoid dropouts by maintaining a patient´s motivation throughout the program, the 3D-MCSTP included a self-assessment notebook and four in-office visits managed by an optometrist (L.M.G.) specialized in neurovisual rehabilitation. The self-assessment notebook included 12 weekly self-assessment tables in which patients or their relatives registered the daily amount of time (minutes) required to perform each exercise at each home-based training session. Thus, indirectly, this notebook was intended to promote patient motivation at the same time that it improved their visual-processing speed, working memory,^56^ and attentional and inhibition of return^57^ brain mechanisms, because they should be able to make more complex stimulus-response associations in the shortest time possible, week-by-week or level-by-level. Accordingly, it should be noted that each exercise methodology facilitated its self-evaluation, because both during the performance of each exercise and at the end of it, patients themselves or their relatives had to correct any possible error before stopping the timer and recording the total time spent performing each exercise correctly. Data about who checked (the patient, relatives, or both) the correct performance of each exercise had to be registered in the self-assessment notebook. Therefore, at the first in-office visit, participants received the neurovisual rehabilitation pack (Fig. 1A.1), which included a box with all neurovisual rehabilitation materials and the first notebook of exercises with the first three levels of difficulty and, they were instructed in performing them. They also were informed that they should choose a table in their home (with a minimum size of 60 × 110 cm) and convert it into their worktable for 12 weeks, at which they could perform the 3D-MCSTP daily with the least possible distractions. Moreover, caregivers were asked to check that patients performed the exercises correctly according to the instructions (mainly the performance of the exercises without head movements), especially at the beginning of the rehabilitation program. The three remaining in-office visits included checking participants’ self-assessment notebook, exchanging the notebook of exercises, and instructing them in performing the following three levels of exercises for the next three weeks of home-based training.

### NTG: Scientific Basis and Procedures

An NTG was included in our study to demonstrate whether it was worth training patients with hemianopia using a customized neurovisual rehabilitation program. It had been reported previously that “[untreated/no-training] controls can only be used when it is possible to ensure that (1) the control group participants are not given the experimental treatment, (2) they cannot obtain it from any other provider, and (3) they cannot obtain any other treatment for the same problem.”^58^^,^^59^ Therefore, in line with these three principles and also for ethical reasons,^59^ our NTG received at the baseline visit a “standard care-advice only” based on a previous hemianopia study.^60^^,^^61^ It consisted of (1) informing patients and their relatives about how a visual field impairment affects the performance of daily activities and (2) advises them to optionally read a book (with the same characteristics as the IG book) and perform a daily ocular motility exercise for 12 weeks of follow-up, which prevented these patients from finding another type of neurovisual rehabilitation on their own. Hence, for them to decide freely and voluntarily to perform the ocular motility exercise at their homes, they received a schematic picture with instructions at the baseline visit (Supplementary Material B). Finally, to have a rough idea about how many patients had chosen to voluntarily perform the ocular motility exercise and the reading task, these data were recorded at the end of the study.

### Assessment Materials and Procedures

**Figure 4.**
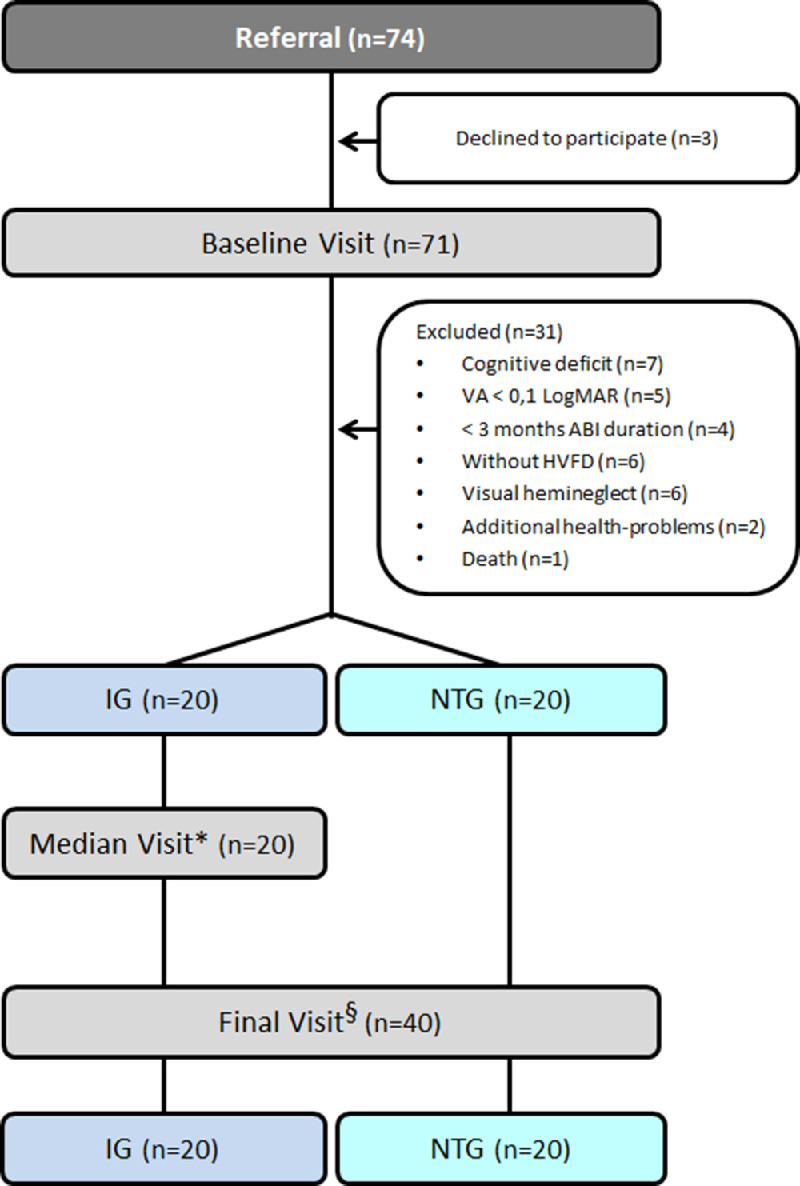
The flow chart of patient recruitment and follow-up assessment visits. *Performed by IG six weeks after the baseline visit, which coincided with the third in-office visit included at the training protocol. ^§^Performed by both study groups at 12 weeks after the baseline visit. VA LogMAR, logarithm of the minimum angle of resolution visual acuity.

Both study groups completed the same assessment procedures under unmasked conditions, before (baseline visit) and after training (final visit) for the 3D-MCSTP effectiveness study, with effectiveness defined as the ability to achieve a specific enhancement^62^ in visual ability/function, QL, and FI, using their dominant hand or the ipsilateral hand if they had hemiparesis (Fig. 4, Table 1). However, only IG participants were assessed at week 6 during a median visit, which coincided with the third in-office visit included in the training protocol for the 3D-MCSTP efficiency study, with efficiency defined as the ability to achieve a specific enhancement in the shortest possible time^62^ in visual ability/ function, QL, and FI, using the same assessment procedures as during the baseline visit and final visit (Fig. 4). Outcome variables for visual ability and visual function assessment were determined by the following assessments.

**Table 1.** Groups Demographic Data

Parameter	IG (n = 20)		NTG (n = 20)		*P* Value
Mean age, years (SD)	59.15	(15.81)	58.65	(17.22)	0.9243^a^
Gender, n (%)					0.0550^b^
Male	15	(75)	8	(40)	
Female	5	(25)	12	(60)	
Mean ABI duration, m (SD)	19.15	(28.07)	23.5	(27.43)	0.2837^c^
Etiology, n (%)					0.3619^d^
Ischemic stroke	12	(60)	10	(50)	
Hemorrhagic stroke	0	(0)	3	(15)	
Tumor	5	(25)	3	(15)	
Intracranial aneurysm	3	(15)	4	(20)	
HVFD, n (%)					1.0000^e^
RHH	7	(35)	7	(35)	
RSHQ	3	(15)	3	(15)	
RIHQ	1	(5)	1	(5)	
LHH	7	(35)	7	(35)	
LSHQ	1	(5)	0	(0)	
LIHQ	1	(5)	2	(10)	
Mean visual acuity (SD)	−0.09	(0.06)	−0.01	(0.07)	0.0004^a^
Hand motor disability, n (%)					1.0000^e^
Yes	10	(50)	11	(55)	
No	10	(50)	9	(45)	

ABI, acquired brain injury; HVFD, homonymous visual field defect; RHH, right homonymous hemianopia; RSHQ, right superior homonymous quadrantanopia; RIHQ, right inferior homonymous quadrantanopia; LHH, left homonymous hemianopia; LSHQ, left superior homonymous quadrantanopia; LIHQ, left inferior homonymous quadrantanopia.

a Student's *t*-test.

b χ^2^ contrast.

c Mann-Whitney U.

d Fisher test.

A computerized visual-processing speed assessment system.^10^ This system can obtain in 15 to 20 minutes (depending of patients’ ability) a minimum of 96 objective values of patients’ reaction times in milliseconds at the time of search by saccadic eye movements and reach by eye-hand coordination mechanisms using four different categories of daily stimuli, which were presented along eight radial positions of their visual field at eccentricities of 10°, 20°, and 30°. The system also registers the number of errors made and the degrees of the head movements during the evaluation using a specific head-tracker system.

Monocular 30-2 perimetry (Humphrey Perimeter, version 4.2 of the system software II series; Carl Zeiss Meditec, Jena, Germany). The perimeter analyzes 76 points in 60° of the visual field (30° from the central fixation target to nasal, temporal, superior and inferior sides). The reliability indexes to consider the results of a monocular perimetry valid were those established by the manufacturer: fixation losses <20% and false-positive and -negative errors <15%. The deviation or mean defect value was used to assess this test, which is defined as the difference between the normal sensitivity expected based on the patient's age (manufacturer's normality database) and the real sensitivity found in the patient examined.

The IReST Test^63^ (Precision Vision (Spanish version), La Salle, IL, USA). This reading performance test includes 10 paragraphs with the same degree of difficulty and linguistic characteristics among them (10-point Times New Roman font) and is presented at a viewing distance of 40 cm. Patients are instructed to read aloud as fast as possible while attempting to avoid mistakes. A different paragraph was used at each of the evaluation visits. The corrected reading performance in words per minute was calculated using the following formula: [(words read − number of errors)/minutes spent reading].

The Benton Visual Retention Test^64^ (Pearson, Madrid, Spain) was used to assess the attentional and retention ability of patients. The test includes 30 sheets measuring 21 × 14 cm divided into three blocks of 10 sheets with geometric drawings in increasing order of difficulty. To avoid the learning effect, patients performed a different block at each evaluation. After visualizing and memorizing each sheet for 10 seconds, patients then drew the original drawing (immediately from memory) on a white sheet of the same dimensions as the original. The objective, considered a variable of measurement, was the total number of correct reproductions made; these were evaluated according to the evaluation standards described in the test manual.

Subjective questionnaires and scales also were used to evaluate the QL and FI that included the Short Form-36,^65^ National Eye Institute Visual Function Questionnaire-25 (NEI VFQ-25),^66^ Goldberg Scale,^67^ Functional Independence Measure^68^ and Pfeffer test.^69^

### Statistical Analysis

Statistical package R version-3.5.1 (R-CoreTeam, 2018, R Foundation for Statistical Computing, Vienna, Austria) was used with a significant value of *P* ≤ 0.05. Intergroup and intragroup effectiveness analysis was carried out between the baseline and final visits. The equality hypothesis of means was evaluated in quantitative variables (Student's *t*-test) and ordinal variables using the Mann-Whitney test. Qualitative variables were evaluated using the χ^2^ contrast or Fisher's exact test. The estimation of change (% effectiveness) for visual ability and function tests was calculated using the following expression: [(value final visit − value baseline visit)/(value baseline visit) × 100]. Moreover, a specific statistical analysis of the visual ability/function variables was performed to control bias because of the fact that the IG was evaluated on one more occasion than the NTG (see also limitations section). This analysis consisted of determining intergroup differences between the median visit of the IG and the final visit of the NTG, using the same previously describe statistical methodology. For QL and FI questionnaires and scales, the effect size was estimated through Cohen's d.^70^ This measure is tabulated and the values between 0 and ±0.2 correspond to negligible effects, between ±0.2 and ±0.49 to small effects, between ±0.5 and ±0.79 to moderate effects, and ≥ ±0.8 to large effects. Positive values represent increases and negative decreases in the score. Student's *t*-test was used to compare values of percent effectiveness between groups.

The IG efficiency analysis of variance with three repeated measures was used in an intrasubject factor (visit), with three levels (baseline visit, median visit, and final visit). Friedman's contrast was used for ordinal variables with repeated measures and the comparisons by pairs of visits by the Wilcoxon's contrast. Alternatively, in the case of qualitative variables, the groups were compared using a contrast of proportions.

Specifically, the global results of the computerized visual-processing speed assessment system^10^ were summarized by a structural equation models analysis (Supplementary Material C).

## Results

Thirty-four of the 74 recruited patients were excluded from the study (three declined to participate and 31 did not meet the inclusion criteria) (Fig. 4). The final study groups included 40 patients, 20 each in the IG and NTG. The main descriptive parameters of both groups are shown in Table 1. Both groups were comparable in terms of the main visual ability and visual function, QL, FI, and baseline variables (*P* > 0.05; Tables 1 and 2).

**Table 2.** Summary of the Main Results from Visual Ability and Visual Function Tests

	Descriptive Data^a^						Percent Change (%)^b^				
	Baseline Visit			Final Visit			Final Visit – Baseline Visit				
	IG (n = 20)	NTG (n = 20)		IG (n = 20)	NTG (n = 20)		IG (n = 20)		NTG (n = 20)		
Visual Ability and Visual Function	Mean/Median (SD)/(IQR)	Mean/Median (SD)/(IQR)	*P* Value	Mean/Median (SD)/(IQR)	Mean/Median (SD)/(IQR)	*P* Value	Mean (SD)	*P* Value Intragroup	Mean (SD)	*P* Value Intragroup	*P* Value Intergroup
Global visual processing speed (ms)	3810.98 (1341.93)	3783.21 (1313.52)	0.9476	2389.22 (664.8)	3885.96 (1742.97)	0.0015	57.34 (19.28)	<0.0001	2.46 (16.08)	0.5015	<0.0001
Seeing hemifield (ms)	3917.84 (1640.60)	3777.63 (1452.84)	0.7763	2317.07 (788.01)	4038.14 (1771.74)	0.0005	38.02 (12.81)	<0.0001	−5.47 (17.67)	0.0910	<0.0001
Blind hemifield (ms)	3980.64 (1443.55)	4407.89 (2214.64)	0.4749	2643.81 (896.97)	4665.39 (2691.96)	0.0041	32.38 (8.18)	<0.0001	−3.95 (17.57)	0.1637	<0.0001

Monocular Perimetry (MD)	−13.63^c^ (4.11)	−12.88^d^ (4.24)	0.5989	−12.14^e^ (3.97)	−12.55^f^ (3.91)	0.7524	−10.12 (15.20)	0.0117	3.88 (9.58)	0.1388	0.0042

Reading performance (wpm)	104.98 (48.27)	107.18 (47.57)	0.8854	113.41 (49.61)	105.02 (51.70)	0.6037	9.88 (10.46)	0.0005	−3.50 (13.63)	0.2651	0.0013

Visual attention-retention (N°-CR)	5.00 (4.00)	6.00 (5.00)	0.5301^g^	7.00 (2.25)	4.00 (4.50)	0.0074^g^	26.67 (19.21)	<0.0001	−2.08 (17.79)	0.6461	0.0001

IQR, interquartile range; MD, mean deviation; wpm, words per minute; N°-CR, number of correct reproductions.

a Lower mean values indicate better performance of the visual processing speed variable. Higher mean values indicate better performance in monocular perimetry, reading performance, and visual attention-retention variables.

b Lower mean values indicate better performance of the monocular perimetry variable. Higher mean values indicate better performance in visual processing speed, reading performance, and visual attention-retention variables.

c n = 1, number of patients eliminated from statistical analysis because of lack of reliability in their measurements.

d n= 3, number of patients eliminated from statistical analysis because of lack of reliability in their measurements.

e n= 1, number of patients eliminated from statistical analysis because of lack of reliability in their measurements.

f n= 2, number of patients eliminated from statistical analysis because of lack of reliability in their measurements.

g Mann-Whitney test.

### 3D-MCSTP Effectiveness Study

Considering effectiveness as the ability to achieve a specific enhancement^62^ in terms of visual processing speed, attention-retention, QL, FI, visual field, and reading performance.

## Main Outcomes

### Visual-Processing Speed

The equality hypothesis of means (Student's *t*-test) showed significant differences between groups at the final visit (*P* = 0.0015). IG patients improved significantly, i.e., with decreased test completion times (milliseconds) in 57.34% (standard deviation [SD] = 19.28; *P* < 0.0001) of their visual-processing speed after 12 weeks of training, whereas the NTG did not improve significantly by 2.46% (SD = 16.08; *P* = 0.5015) in this variable after 12 weeks of follow-up (Table 2). Both groups’ visual-processing speed changes occurred on both hemifields equally at the end of the study (being significant improvements in IG and no significant worsening in NTG; Table 2). That is to say, no statistically significant differences (neither for IG *P* = 0.1066, nor NTG *P* = 0.7887) were found between reaction times means changes corresponding to the seeing side (IG: 38.02%, SD = 12.81, *P* < 0.0001; NTG: −5.47%, SD = 17.67, *P* = 0.0910) and the blind side (IG: 32.38%, SD = 8.18, *P* < 0.0001; NTG: −3.95%, SD = 17.57, *P* = 0.1637). Changes in seeing and blind side performance were significant between groups (*P* < 0.0001) at the end of the study. Both groups showed no significant differences between seeing and blind side visual-processing speed performance (descriptive data means values) neither at baseline visit (IG: *P* = 0.8984; NTG: *P* = 0.2949) nor at final visit (IG: *P* = 0.2287; NTG: *P* = 0.3903). At the baseline visit and final visit, both groups moved their heads along the horizontal meridian no more than 3.5° (range 0.35°–3.37°) and made no more than one mistake (range 0–1) during the visual-processing speed assessment test. The statistical analysis of bias control showed significant intergroup differences (*P* = 0.0013), with a visual-processing speed improvement in the IG of 21.73% (SD = 18.94; *P* = 0.0001) when their median visit and baseline visit results were compared.

### Attention-Retention

The equality hypothesis of means (Student's *t*-test) showed significant differences between groups at the final visit (*P* = 0.0074). The IG also had a significant improvement of 26.67% (SD = 19.21; *P* < 0.0001) in the number of correct reproductions; while the NTG had a worsening of −2.08% (SD = 17.79; *P* = 0.6461) (Table 2), which was not significant. The statistical analysis of bias control showed nonsignificant intergroup differences (*P* = 0.5063), with the IG attention-retention improvement of 2.78% (SD = 24.15; *P* = 0.6129) when their median visit and baseline visit results were compared.

### QL and FI

**Figure 5.**
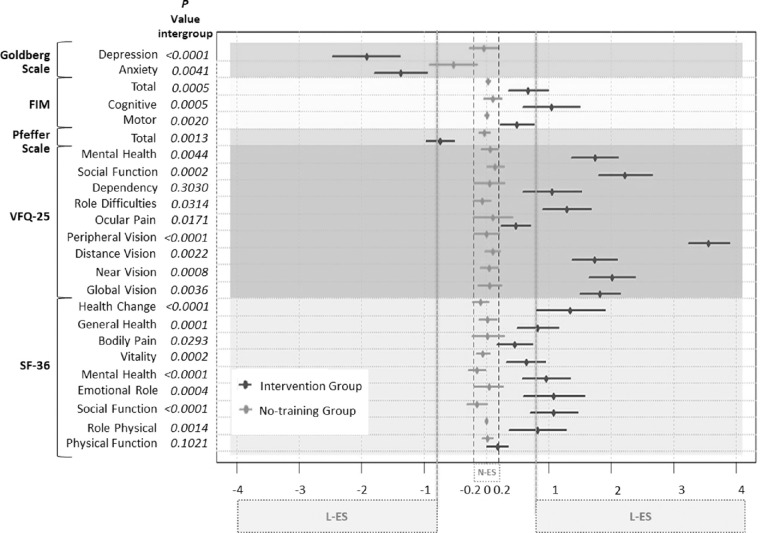
The Cohen's d summary chart for the dimensions of the five questionnaires and scales of QL and FI used. The no-training group shows changes with negligible effect sizes (*N-ES*) (range 0 to ±0.2) in 96% of the evaluated dimensions. The intervention group shows changes with large effect sizes (*L-ES*) (≥ ±0.8) in 75% of the dimensions evaluated. Intergroup *P* values have been represented in *italic font*. Higher negative values representing more improvements are seen only for the Goldberg scale and Pfeffer scale. In the other questionnaires and scales, the positive values represent improvements. FIM, Functional Independence Measure; VFQ-25, Visual Function Questionnaire-25; SF-36, Short Form-36.

Regarding the QL and FI results (Fig. 5), the NTG showed negligible mean effect sizes in 96% of all dimensions analyzed; the anxiety dimension of the Goldberg Scale was the only one that showed a moderate change effect. In contrast, the IG showed large change effects in 75% of the evaluated dimensions, especially in peripheral vision. Moreover, there were significant differences between groups in 92% of all dimensions studied (*P* < 0.05; Fig. 5).

## Secondary Outcomes

### Visual Field

The equality hypothesis of means (Student's *t*-test) did not show significant differences between groups at the final visit (*P* = 0.7524). Considering the mean deviation (decibels [dB]) between both eyes, the IG patients improved significantly by 10.12% (SD = 15.20; *P* = 0.0117) of their visual field after 12 weeks of training, whereas the NTG worsened but not significantly by 3.88% (SD = 9.58; *P* = 0.1388) of this variable after 12 weeks of follow-up (Table 2). The statistical analysis of bias control showed significant intergroup differences (*P* = 0.0123), that is, a IG visual field improvement of −9.43% (SD = 17.12; *P* = 0.0372) when their median visit and baseline visit results were compared.

### Reading Performance

The equality hypothesis of means (Student's *t*-test) did not show significant differences between groups at the final visit (*P* = 0.6037). However, the IG had a significant improvement of 9.88% (SD = 10.46; *P* = 0.0005) in their reading performance (words/minute); while the NTG had a worsening of -3.50% that was not significant (SD = 13.63; *P* = 0.2651) (Table 2). The statistical analysis of bias control showed significant intergroup differences (*P* = 0.0038), with the IG reading performance improvement of 7.54% (SD = 8.45; *P* = 0.0008) when their median visit and baseline visit results were compared.

### 3D-MCSTP Efficiency Study

Considering efficiency as the ability to achieve a specific enhancement in the shortest possible time^62^ in terms of visual ability/function, QL, and FI.

### Visual Ability and Function

**Figure 6.**
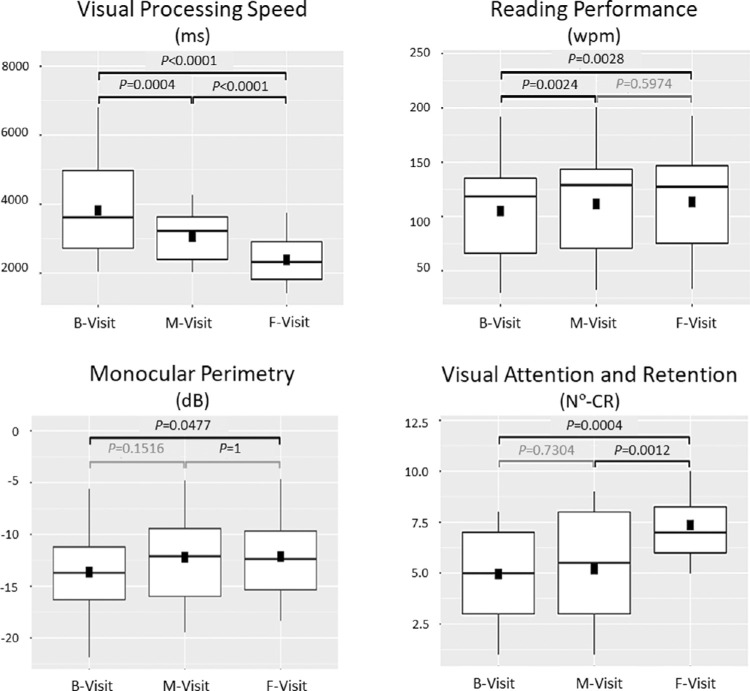
Efficiency analysis results in terms of visual ability and function. The intervention group boxplot efficiency analysis results for the principal visual ability and function variables at the baseline visit (*B-visit*), median visit (*M-visit*), and final visit (*F-visit*). The *square* represents the mean, and the *horizontal line* the median. The *P* values were obtained by statistical analyses of equal visit contrast. wpm, words per minute; N°-CR, number of correct reproductions.

The IG had a significant improvement in the visual-processing speed (95% confidence interval [CI] milliseconds [ms]: −1,092.70; −394.52, *P* = 0.0008) and reading performance (95% CI [words/minute]: 3.03; 9.77, *P* = 0.0024) after the first six weeks of training (Fig. 6). However, the visual-processing speed continued to improve significantly between the median visit and final visit (95% CI [ms]: −767.84; −588.45, *P* < 0.0001).

The variable visual attention-retention did not improve significantly until the second period of training (weeks 6–12) (95% CI [number of correct reproductions]: 1; 3, *P* = 0.0012) (Fig. 6).

The monocular perimetry only showed significant differences after 12 weeks of training (95% CI [mean deviation, dB]: 0.31; 2.60, *P* = 0.0477) (Fig. 6).

### QL and FI

The 3D-MCSTP was more efficient at 12 weeks after its implementation than at 6 weeks, both in the QL and FI. Thus, if the 3D-MCSTP had lasted only six weeks, no improvements would have been found in the QL regarding the dimensions of the physical role, emotional role, vitality, bodily pain, and ocular pain, which began to show significant increases (improvement; *P* < 0.05, Supplementary Material D, Supplementary Material E and Supplementary Material F) from the median visit (at week 6 of training) or at the final visit (at week 12 of training).

### Level of Compliance

All IG patients exceeded the minimal daily neurovisual rehabilitation regimen (mean, 159 sessions; range, 126-168), and they could perform exercises at the highest level of difficulty (level 12: 25.79 ± 5.96 minutes) in similar times (*P* = 0.1497) to those in which they performed exercises at the minimal level (level 1: 24.64 ± 8.03 minutes). Forty-five percent of IG patients performed home-based exercises without assistance, whereas the other 55% performed them with the assistance of their caregivers. Ninety percent of IG patients met the minimal reading pattern.

Fifty percent of the NTG patients reported that they performed the voluntary ocular motility exercise in an average of 12.08 ± 5.10 minutes 5 days/week; the other 50% reported never performing it. Forty percent of the NTG patients reported that they read daily, whereas the remaining 60% said they had not read anything during the 12 weeks of the study.

## Discussion

The study results are promising and strongly suggest that the 3D-MCSTP facilitates improvement of the functional prognosis of a specific sample of patients with hemianopia regarding complex visual-brain processing mechanisms, QL, and FI. In addition, the 3D-MCSTP proved to be motivating and it had very high compliance level over 12 weeks, because all IG patients exceeded the minimal daily home-based neurovisual rehabilitation regimen and they attended all four in-office visits included in the training protocol. The use of a self-assessment notebook had three positive aspects, in that it provided objective information missing from previous studies, as de Haan and collaborators mentioned in their meta-analysis^71^; it can motivate patients to self-assess daily if they can perform exercises in shorter times; and finally it provided information about improvements in the patients’ visual-processing speed, since it showed that they performed exercises at level 12 with times similar to those of level 1. However, its main limitation was its dependence on the degree of commitment of the caregivers and patients. Therefore future studies should replace the self-assessment notebook with more objective evaluation tools principally to reduce the degree of dependence of those patients who needed caregivers help to record the times they took to perform each exercise.

IG patients had the most significant improvements in the visual-processing speed by reducing their reaction time by about 55% more than the NTG after 12 weeks of training. According to the visual-processing speed assessment methodology used,^10^ these results suggested improvements in complex brain-processing mechanisms (Fig. 1), which are fundamental for performing everyday multitasking activities. Moreover, the results agreed with recent neuroscientific studies that advocated the benefits of visuomotor multitasking training using 3D real-world objects instead of 2D images, because of the activation of the four cerebral lobes, brainstem, and cerebellum, and at the same time the increasing information about processing speed in the human prefrontal cortex.^1^^,^^20^^–^^22^^,^^72^ Our results also suggest that hemianopia patients included in our study had globally affected their visual-processing speed because, at the beginning and at the end of the study no significant differences were found for this variable when the results for the seeing and the blind hemifields were compared, in either group. These results could be related to the fact that patients with visual hemineglect were not included. Furthermore, they are in accordance with a previous study carried out for our group^10^ and with other authors, who by using eye tracker systems did not find significant differences in amplitude or frequency of saccades between both hemifields.^73^^,^^74^ In addition, our results showed that the 3D-MCSTP significantly improves visual processing speed on both hemifields (seeing and blind) equally. Therefore these results suggest that visual abilities of both hemifields should be trained equally in hemianopia patients without hemineglect. However, due to the small sample of our study and its limitations, further studies are needed. In fact, Roth et al.^17^ only found improvements for the blind side on digit-search reaction times and natural search reaction times, after their computerized compensatory training. However, their reaction times assessment methods were quite different than ours. Also, they did not objectively control for patients head movements during these assessments. Furthermore, the IG visual-processing speed results do not seem to result from a learning effect of the assessment method, in that the patients performed the test one more time (median visit) than the NTG, because the IG reaction time at the median visit was already about 19% better than the NTG reaction time at the final visit. In addition, there was a longer period of time between each evaluation visit (1.5 months). Certainly, the IG median visit was a limitation in our study (see also Limitations section), but at the same time, it facilitated our reaching important conclusions about the 3D-MCSTP efficiency. For example, regarding the visual-processing speed, the median visit results showed that 12 weeks of training rather than six weeks, as the main current compensatory training programs suggests,^17^^,^^18^ improved the efficiency of our training program. This allowed IG patients to improve their reaction times about 35% more than if their training lasted six weeks. However, only two published computerized compensatory neurovisual rehabilitation training programs with three methodologic aspects are available that facilitates an approximated comparison between them and our 3D-MCSTP. First, they rely on an NTG. Second, its training should be performed by the patients’ daily at their own homes over six weeks.^17^^,^^18^ Third, they presented their patients' reaction times improvements as percentages of change. In contrast to our current methods, their reaction time computerized evaluation methods were similar^18^ to those of training or exactly the same.^17^ Accordingly, our IG reaction time improvements at the median visit were about 9% higher than those reported by Aimola et al.^18^ and about 25% lower than those of Roth et al.^17^ However, when those authors used non-computerized assessment methods (Evaluation Test of Activities of Daily Life^18^ and “natural search test”^17^), they did not find significant reaction time changes^18^ or they found significant reaction time improvements only about 1% higher^17^ than the current improvements at the median visit. They argued that their noncomputerized assessment methods required activation of more complex brain-processing mechanisms (eye-hand coordination, attention, executive functions, etc.) than those required to search simple stimuli on a computer screen. Therefore, our 3D-MCSTP may be about 34%^17^ and 45%^18^ more effective than those previous computerized compensatory training programs, since the IG improved their visual-processing speed by 57% at the end of training. Furthermore, the IG improved about 29% more than the NTG, which in the attention-retention variable even worsened by about 2% at the end of the study. These attention results exceeded previous studies that failed to identify objective improvements in these tasks.^17^^,^^18^ Moreover, they are in accordance with previous studies that found brain attention network improvements after minimal training periods of 12 weeks. For example, MacLean et al.^38^ found that 12 weeks of intense meditation training can improve performance on tasks of perceptual discrimination and sustained visual attention. Furthermore, Lawton^39^ and Lawton and Shelley-Tremblay^40^ found significantly attention improvements, processing speed, reading fluency, and working memory, in dyslexics’ patients after 12 weeks of specific perception attention therapy.

Regarding reading performance, the IG had an improvement of about 13% higher than the NTG, which worsened by about 3.5%, although an efficiency study verified that the IG did not improve significantly from the median visit to the final one. Thus, by including a specific reading task in the training protocol, our approach improved over previous approaches (except for that of Aimola et al.^18^), that did not include specific reading tasks in their training. Our approximately 7.5% improvement in reading performance obtained between the baseline visit and median visit was about 11% lower than the improvement reported by Aimola et al.^18^ in the same training period. Nevertheless, this comparison must be interpreted with caution because their nonstandardized reading test was considerably less difficulty (its font size was 14 points and its paragraphs were justified with a double separation between reading lines) than the standardized IReST Test.^63^ Hence, our reading-performance results suggested perfecting the guideline and read-aloud exercise in future studies to achieve greater reading-performance efficiency. Because the IG improvement found in our study regarding this variable has low clinical significance, the equality hypothesis of means (Student's *t*-test) did not show statistically significant differences between groups during the final visit.

Regarding the visual field variable, the IG improved about 14% on monocular perimetry compared to the NTG that worsened about 4% at the end of the study. This IG visual field improvement has low clinical significance, because the equality hypothesis of means (Student's *t*-test) did not show significant differences between groups at the final visit with regard to this variable. Moreover, the IG monocular perimetry improvement should be interpreted with caution for three reasons. First, they could be related to spontaneous recovery. Although this seems unlikely, because there were no significant differences between groups related to acquired brain injury duration and even the NTG had 10% more patients than the IG with less than six months of acquired brain injury duration. Second, the improvement may have been related to a learning effect,^75^ because the IG was exposed to this test one time more than the NTG. However, at the median visit, the IG had already improved significantly by about 13% more than the NTG in monocular perimetry at the final visit. Third, the improvement may have been related to the fact that all these tests required high attentional capacity and fast reaction times. Consequently, these visual field improvements may have been related directly to previous visual-processing speed and attention-retention improvements discussed. However, future studies that include, for example, functional magnetic resonance assessment methods should corroborate this hypothesis.

Finally, IG showed significant improvements against NTG in 92% of the QL and FI dimensions studied. The NTG showed negligible change effect in all five test dimensions, except for anxiety, showing a moderate change effect probably because 50% of the NTG patients performed the optional ocular motility exercise. And, although this voluntary exercise has been ineffective in terms of visual ability and function, it may have contributed to reduce anxiety. Furthermore, IG's objective improvements in terms of VPS and visual attention-retention were accompanied by significant QL and FI subjective improvements. On the other hand, other computerized hemianopia training programs like the one by Aimola et al.^18^ reported that subjective everyday improvements (using the NEI VFQ-25) were not accompanied by objective visual ability data (using the specific evaluation test of activities of daily life). Roth et al.^17^ found that its IG only reported QL significant improvements in social-relationships domain (using the World Health Organization questionnaire on quality of life). These discrepancies with our program could be related to a more practical approach and to the fact that it lasted a longer time. Accordingly, the 3D-MCSTP showed greater efficiency in terms of the QL and FI at 12 weeks of its implementation.

### Limitations and Future Lines

The inclusion of the median visit in the IG assessment protocol was a limitation of our study; because ideally, both groups should complete the same number of assessment visits to principally avoid the learning effect phenomenon. This phenomenon implies that certain parameters of an assessment test may experience some improvement as the patient's experience in performing the tests increases.^76^ It is an important issue in many psychophysical tests, such as the visual field testing using Humphrey perimeter.^75^^,^^77^ However, it was absolutely necessary to include the median visit in the IG assessment protocol to compare and determine the outcome if the training was extended for an additional six weeks more than the main compensatory hemianopia training programs with training periods of six weeks.^17^^,^^18^ Furthermore, no-training controls are particularly vulnerable to problems associated with treatment fidelity procedures and allegiance biases.^59^ Accordingly, the idea that our NTG also should complete a median visit was rejected, both to avoid dropouts and to have a NTG that closely resembled the current active protocols that national health systems usually apply to patients with hemianopia.^24^^–^^26^ In our study, the visual ability and function variables were the most likely to be affected by the learning effect phenomenon. Thus, although in our study there was a long period of time between each assessment visit (1.5 months, which could contribute to diminishment of the learning effect phenomenon), a specific statistical analysis of the visual ability/function variables was performed to test the effects of change between the median visit of the IG and the final visit of the NTG. That is, when both study groups had been subjected to the assessment test an equal number of times (two visits). In this manner, it was possible to objectively check that the IG at the median visit had significant improvements compared with the NTG at the final visit, in three of the four variables of visual ability and function studied (visual-processing speed, visual field, and reading performance). Hence, these results could be related to a greater extent with the performance of the 3D-MCSTP than with a learning effect phenomenon. However, future studies should corroborate our results by improving the evaluation protocols, e.g., a protocol with an equal number of IG and NTG assessment visits. Moreover, in the future, it would be convenient to compare our training approach with other existing compensatory neurovisual rehabilitation programs, for example, through crossover studies. It also would be interesting to combine its application with more specific therapies that are effective for activating mechanisms of neuroplasticity after an acquired brain injury, such as transcranial direct current stimulation.^78^ Finally, long-term effectiveness studies and randomized controlled clinical trials with masked assessment procedures should follow the current report given the encouraging results attained.

## Supplementary Material

Supplement 1

Supplement 2

Supplement 3

Supplement 4

Supplement 5

Supplement 6
