# Supplementary material for: Multitasking Compensatory Saccadic Training Program for Hemianopia Patients: A New Approach With 3-Dimensional Real-World Objects
Source: Transl Vis Sci Technol. 2021 Feb 5;10(2):3. doi: 10.1167/tvst.10.2.3 (PMC7873505; doi:10.1167/tvst.10.2.3)
Supplement: Supplement 1 [file tvst-10-2-3_s001.pdf]

Supplementary Material A

Summary of the main materials selected for designing the eight multitasking exercises:

- Exercise (E) 1: one Spanish pack of 40 cards divided into four families (golds, cups, swords, and clubs) numbered from 1 to 12 (Fournier, Álava, Spain).
- E 2: one game board with 10x10 grids (labeled A-J on the y-axis and, 1-10 on the x-axis) and 65 insertion-pivots of battleship-game (Falomir Juegos, Valencia, Spain).
- E 3: 21 Bingo cards and 90 pieces numbered from the 1-90 of Bingo Lotto game (Falomir Juegos). Sixty circular plastic pieces were used as number markers.
- E 4: 28 pieces of Domino (Fournier). Each piece was rectangular and its anterior face was divided into two squares marked with no to six black dots on a white background.
- E 5: 100 plastic pieces with letters of the Intelect Luxe game (Falomir Juegos).
- E 6: one folding-grid for inserting pieces according to pattern sheets and 42 circular plastic pieces (21 red, 21 yellow) of the Line UP 4-game (Family Fun, Daytona Beach, FL, USA).
- E 7: one red marker pen and pattern copy sheets.
- E 8: seven geometric plastic figures of Tangram (Pavillon, Toys R Us, Singapore): 5 triangles, 1 square, and 1 parallelogram (in other words, 2D copy pattern to 3D pattern reproduction).
