# Supplementary material for: Multitasking Compensatory Saccadic Training Program for Hemianopia Patients: A New Approach With 3-Dimensional Real-World Objects
Source: Transl Vis Sci Technol. 2021 Feb 5;10(2):3. doi: 10.1167/tvst.10.2.3 (PMC7873505; doi:10.1167/tvst.10.2.3)

Supplementary Material B

Written instructions and schematic of the voluntary ocular motility exercise received by the no-training group at the baseline visit:

“You have to cut out 15 3x3-cm squares of paper and number them from 1 to 15. On an empty wall of your home measuring 50x100 cm, you must place these squares of numbers in random positions (with an approximate separation of 20 cm between them). You should sit 65 cm from the wall and look at the central square located at the point of intersection of the two diagonals of the created rectangle. Because the boxes with numbers are independent cards, they can be exchanged manually. Six possible sequences of eye movements could be performed, i.e., even or odd numbers in ascending or descending order and all numbers in ascending or descending order. Moreover, you should perform the ocular motility exercise in the shortest possible time by means of saccadic eye movements without head movements and maintaining a constant distance from the wall.”

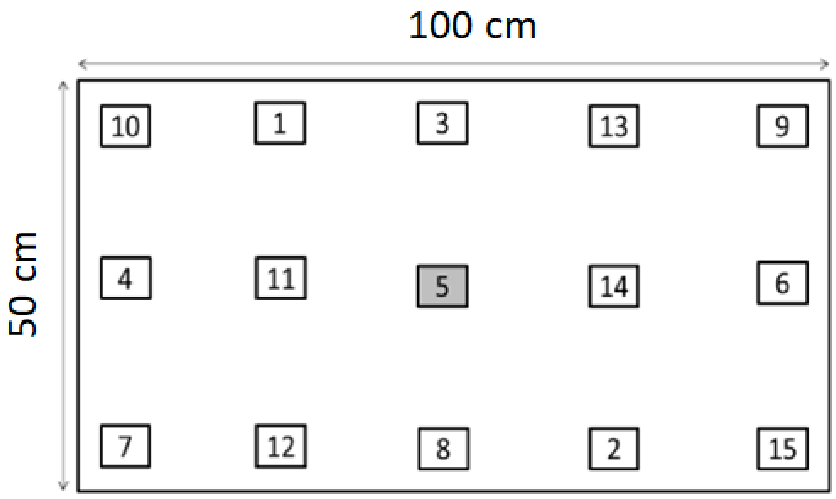

Supplement: Supplement 2 [file tvst-10-2-3_s002.pdf]
