# Supplementary material for: Multitasking Compensatory Saccadic Training Program for Hemianopia Patients: A New Approach With 3-Dimensional Real-World Objects
Source: Transl Vis Sci Technol. 2021 Feb 5;10(2):3. doi: 10.1167/tvst.10.2.3 (PMC7873505; doi:10.1167/tvst.10.2.3)
Supplement: Supplement 3 [file tvst-10-2-3_s003.pdf]

Supplementary Material C

Specific statistical analysis for results of the computerized visual-processing speed assessment system: Considering the values of the complete study sample (40 individuals), structural equation models are used to summarize in a single variable the 96 visual-processing speed measures of RT that the system recorded for each individual. In this way, we used the package Iavaan's R[1] (University of Ghent, Belgium) for the model adjustment and the package semTools[2] (University of Kansas, Lawrence, USA) for the reliability of latent variables. Because of a very strong positive correlation between the 12 evaluated scenarios (3 eccentricities and 4 categories of daily-life stimuli), the variable RT was modeled and a factorial hierarchical model of structural equations was developed with four factors that make up a single variable, referred to as visual-processing speed. To compare this visual-processing speed summary variable, the same methodology was used, as explained previously for variables of ability and visual function.

Supplemental References

1. Rosseel Y. lavaan: An R package for structural equation modeling. *J Stat Softw*. 2012;48:1-36.
2. Contributors2015 S. semTools: Useful tools for structural equation modeling. 2015. http://cran.r-project.org/package=semTools.
