# Supplementary material for: Multitasking Compensatory Saccadic Training Program for Hemianopia Patients: A New Approach With 3-Dimensional Real-World Objects
Source: Transl Vis Sci Technol. 2021 Feb 5;10(2):3. doi: 10.1167/tvst.10.2.3 (PMC7873505; doi:10.1167/tvst.10.2.3)

Supplementary Material F

Efficiency analysis results for the Pfeffer, FIM and Goldberg scales. The intervention group boxplot efficiency analysis results at the baseline visit (B-visit), median visit (M-visit), and final visit (F-visit). The square represents the mean and the horizontal line the median. The *P* values were obtained by statistical analyses of equal visit contrast.

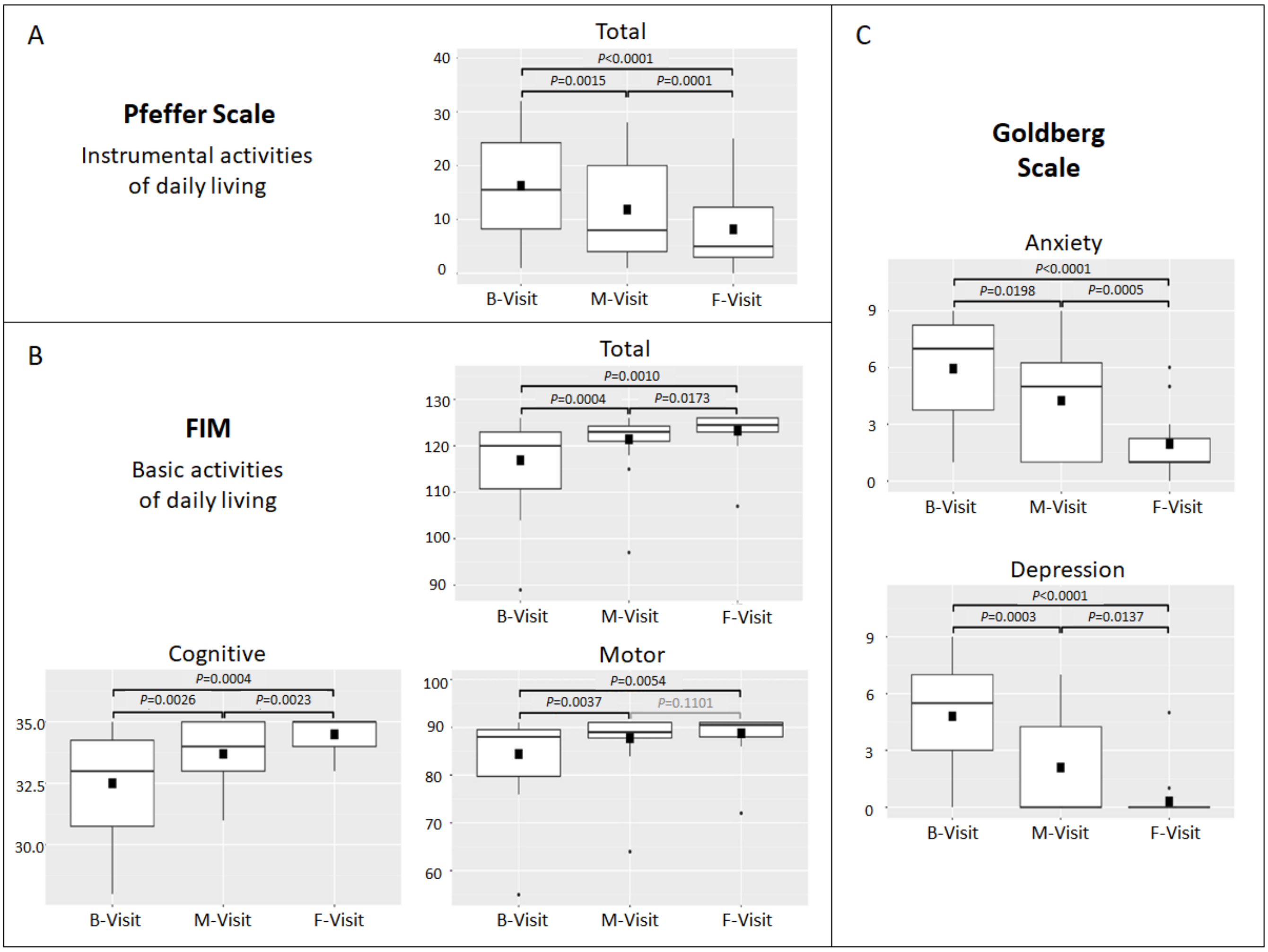

Supplement: Supplement 6 [file tvst-10-2-3_s006.pdf]
